# A stewardship approach to shaping the future of public health supply chain systems

**DOI:** 10.9745/GHSP-D-14-00123

**Published:** 2014-12-02

**Authors:** Alan Bornbusch, Todd Dickens, Carolyn Hart, Chris Wright

**Affiliations:** a United States Agency for International Development, Washington, DC, USA; b PATH, Seattle, WA, USA; c John Snow, Inc, Washington, DC, USA

## Abstract

Guiding Principles: (1) Governments should see themselves as stewards of supply chains, providing vision, guidance, and oversight, not necessarily as operators of supply chains. (2) Governments should not be afraid to leverage the multiple supply chain actors and diverse options available; these can be woven into a coherent, integrated system, providing flexibility and reducing risk. (3) Governments will need new skills in leadership, regulation, market research, contract design, oversight of outsourced providers, financial analysis, and alliance-building.

Picture this: *You are the Minister of Health, responsible for supporting the health of your citizens. Your mandate—your business—is to make sure their needs for services, supplies, and information are met to maintain or recover their health and to live productively in their communities. Lives depend on it, the economy depends on it, and your country depends on it. Part of that mandate is ensuring people can access affordable, quality drugs and other health products, but this is difficult and costly. The citizens you serve—your customers—are from every socioeconomic level and are scattered all over the country, many in very difficult-to-reach areas. And there are larger trends afoot that pose challenges and opportunities for you. How will you lead the effort to ensure an effective supply chain system?*

The global health community has until recently focused much of its attention on achieving relatively near-term goals, such as the 2015 Millennium Development Goals and the Family Planning 2020 goals. However, there is now growing interest in achieving longer-term end games that look a generation into the future, such as attaining universal health coverage, achieving an AIDS-free generation, and ending preventable child and maternal deaths. For those concerned with ensuring access to health commodities, these longer-term visions require a hard look at the supply chain systems of today: How well have they adapted to today's realities? And what must begin now in order to equip them to take advantage of future opportunities and to meet future challenges?

In the commercial sector, every successful enterprise asks similar questions. The answers begin with *knowing your business* and knowing that *your supply chain strategy serves your business strategy*. Taking a business approach means: Understanding your customers' needs and the resources you have available to meet those needs. Understanding your options for supply chain services. Knowing when it is in your comparative advantage to develop in-house capabilities and when to seek those capabilities elsewhere. Contracting with those that have the expertise you need and negotiating rates that provide good value and good services. Managing those contractors and monitoring customer satisfaction. Staying flexible and changing what does not work.

Our view is that this business approach can serve the public health sector as well as it serves the commercial sector. Incorporating it into the broader stewardship responsibilities of government is necessary if public health supply chain systems are to effectively adapt to changing development contexts.

## CHANGING DYNAMICS

In just the last decade, low- and middle-income countries (LMICs) have seen significant economic growth,^1^ with many experiencing economic transitions. This growth has presented opportunities for health financing, expansion of the retail market, and increases in private-sector logistics capacities. The mass penetration of mobile telecommunications is powering a revolution in access to information and enabling better management and greater transparency in supply chain systems. Changing demographics are helping to drive these developments, with continued population growth, a younger population eager for opportunity, and increased urbanization that is concentrating markets. Within the public health sector, the traditional emphasis on communicable diseases has yielded significant gains, while looming priorities will change to address noncommunicable diseases such as cancer, diabetes, asthma, and cardiovascular disease that disproportionally affect LMICs.^2^ Decentralization of government services has increased the complexity of health services with a multitude of new stakeholders, financing options, and decision-makers. New donors and global initiatives have expanded funding sources. Health care consumers, civil society, and development partners are all demanding better performance and cost-effectiveness.

Meanwhile, configuration and management of public health supply chain systems has changed only gradually. These systems strain under the vastly increased volume of products brought by billions of dollars' worth of investments in vaccines, medicines, and supplies to prevent, diagnose, and treat diseases, support family planning programs, and more.^3^ Some investment has focused on strengthening existing in-country supply chains and creating alternative supply channels to compensate for under-performing systems or to respond to larger reforms in government services or the health sector. These investments have prevented collapse and have yielded gains in overall performance in terms of commodity availability. Millions of lives have been improved, and millions of deaths averted. However, these performance gains are tenuous, as are the health outcomes, unless public health leaders take a more business-like approach to challenge the status quo and ask of today's supply chain systems: “Is what we have today working as well as it needs to, and will it work for tomorrow?”

## NEW OPPORTUNITIES, NEW PERSPECTIVES

The people responsible for public health supply chain systems—working in ministries, donor agencies, nongovernmental organizations (NGOs), technical agencies, etc—must expand their perspectives of their own roles as well as of the mission and composition of the supply chain systems they support. There are 3 guiding principles to keep in mind:

### 1. A government's role is one of stewardship in achieving common development goals.

**Figure 1.**
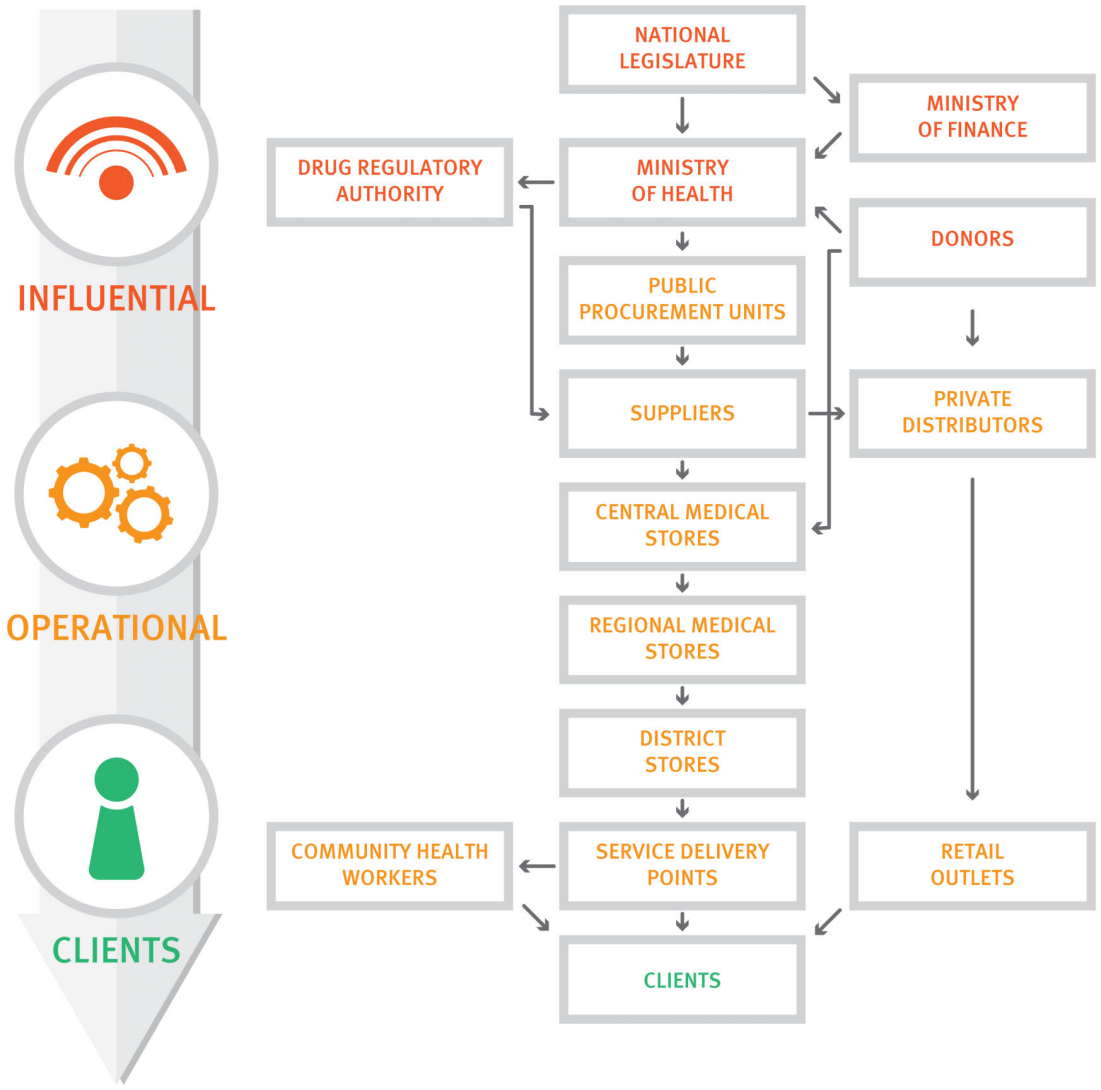
The Traditional Centralized Government-Operated Supply Chain

Governments in particular must understand first and foremost that their core competency is *not* in operating supply chains, which has been their traditional role in centralized systems (Figure 1). Instead they must see themselves as *stewards* providing vision, guidance, and oversight to ensure that supply chains achieve results—serving the needs of customers to improve and maintain people's health. Stewardship does not require direct control of services and facilities; rather, stewards are responsible for engaging and orchestrating different partners to achieve common development goals.

Governments' core competency is not in operating supply chains but in being stewards of supply chains to ensure they achieve results.

### 2. Multiple players and diverse supply chain options can now contribute to public health outcomes.

**Figure 2.**
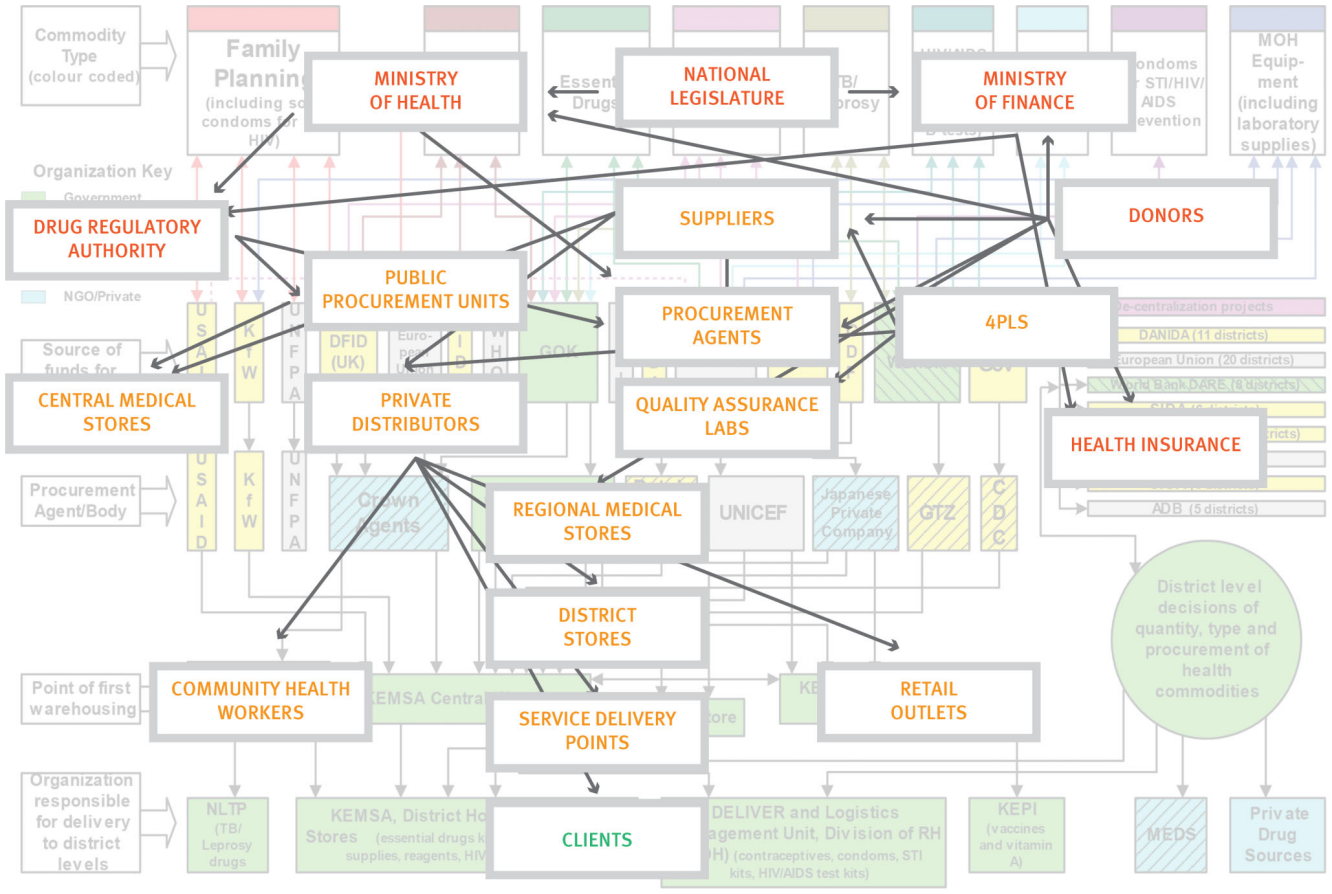
Public Health Supply Chain Systems Today Comprise an Ecosystem of Operational and Influential Actors Color coding as in Figure 1 (red, influential; orange, operational; green, clients). Source: Adapted from Kinzett and Gelfed (2001).^14^

**Figure 3.**
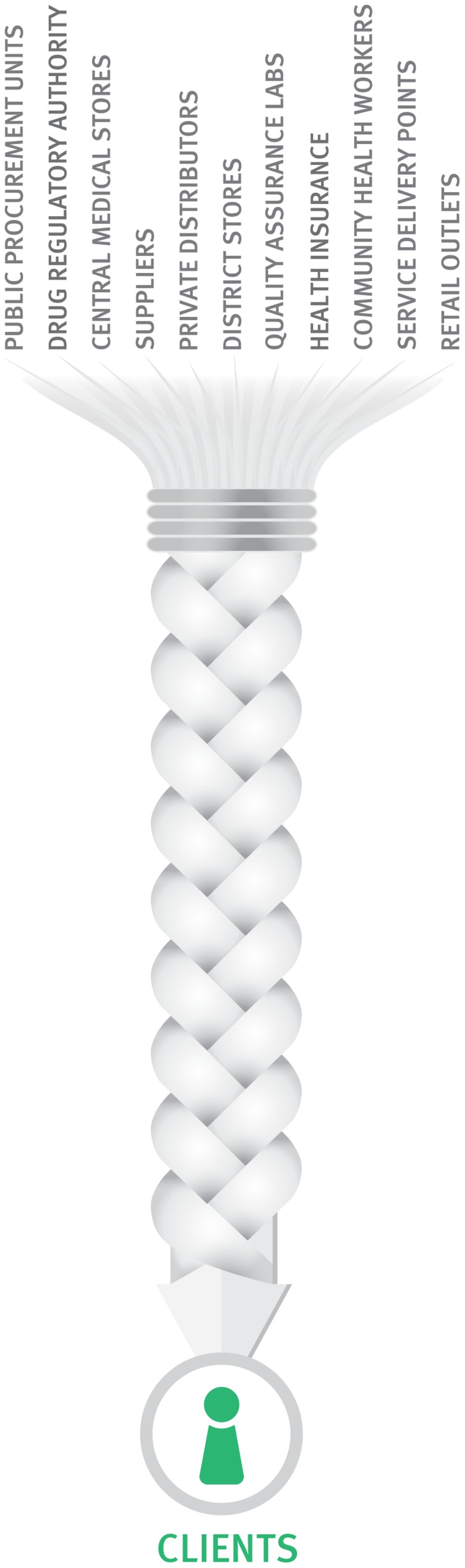
An Integrated Supply Chain System In an integrated supply chain system, people, functions, levels, and actors are linked and managed as an interconnected system.

Stewards must expand their concept of what “supply chains” look like and embrace an increasing diversity of players. Traditional government-operated public health supply chains are an oversimplification and are becoming a thing of the past. In most countries today, the reality is that public health supply chain *systems* encompass multiple supply chains and involve a multi-sectoral range of public, private, faith-based, and NGO facilities and distributors; diverse operational agencies and practices; and people from many organizations and professions (Figure 2). Such complexity can become overwhelming but when well-understood (viz the overlay in Figure 2) and managed, these diverse supply chains and supply chain actors can be woven into a rationally integrated system (Figure 3). This can give stewards flexibility and prudent redundancy in funders, suppliers, distributors, procurement arrangements, and even in quality assurance, reducing risks of supply disruption and better serving all customers.^4^

Public health supply chain systems today encompass multiple supply chains and diverse players.

### 3. Supply chains support broader public health outcomes.

Stewards must understand the intended impact of public health supply chains not simply in terms of distributing products but also in improving health outcomes and even broader development goals, such as increasing productivity and reducing poverty. The cost-benefit analysis of alternative supply chain models should expand to take into account these broader “bottom line” considerations.

## EVOLVING TOWARD STEWARDSHIP, 3PLs, AND 4PLs

Ministries of health have traditionally operated public-sector supply chains through central medical stores and ministry motor pools.^5^ The norm has been control of supply chain *functions* through ownership of supply chain *assets*—people, warehouses, vehicles, etc— in order to fulfill the social and political mandate of making medicines and other health commodities available. However, many governments have neither the expertise to operate efficient supply chains nor the career structures to enable professionalism and promote performance among supply chain workers. Yet the public sector is often reluctant to outsource to third-party logistics providers (3PLs) in the private sector, fearing loss of control or perceiving higher cost, conflict of interest, or misaligned motives.^6^ In addition to those fears, a very real impediment to effective outsourcing is a lack of contract management capacity within government. But just as supply systems need to evolve—from ad hoc activities to more organized approaches and finally to well-integrated systems^7^—stewards of the health sector must also evolve with them.

Outsourcing is increasingly common throughout LMICs for functions such as storage, transport, and procurement, and there are numerous instances of successful private-sector engagement in public health supply systems. Commercial or NGO 3PLs and distributors supply both public and private facilities in Kenya, Malawi, Mozambique, Nigeria, South Africa, Tanzania, Uganda, Zimbabwe, and other countries. For procurement, many governments use services offered by UN agencies and private firms. A fourth-party logistics (4PL) strategy,^8^ for the design, optimization, and operation of supply chains, is increasingly recognized as a good investment in the public sector; 4PLs function as system integrators that coordinate quantification, plan and conduct procurement, and manage freight forwarders, customs clearance, and other 3PLs—essentially taking responsibility for end-to-end supply chain operations on behalf of a client.

Outsourcing supply chain functions is increasingly common in low- and middle-income countries.

At the same time, public agencies can or must fulfill some key roles. For example, e-procurement services for health commodities have been established in Chile^5^ and Indonesia,^9^ where the health minister credits the new service with reducing prices by 40%.^10^ Procurement has been decentralized in Bolivia, Colombia, and Ecuador, but the ministry of health (or its equivalent) prequalifies suppliers based on quality and reliability, and local/regional governments are allowed to procure only from prequalified suppliers.^11^ Greater autonomy of central medical stores, outsourced management, or competition from alternative agencies has improved customer service in Botswana,^5^ Burkina Faso,^12^ Chile, and Uganda.^5^ Among their many important responsibilities, national drug regulatory authorities play an important role in quality assurance (QA) for pharmaceuticals in both the public and private sectors. But where capacity is limited, functions like QA testing can be outsourced, as has been done in Tanzania for some locally procured and distributed medicines.^13^

Effective stewardship of a more diverse supply ecosystem requires not only new perspectives on roles but also new skills and capacities. These include the leadership to articulate a common vision, effective regulation, a robust capacity for market research, contract design, award and management and oversight of outsourced providers, expertise in modeling and financial analysis, and adept alliance-building among partners. Good stewardship also requires commitment to good governance—transparent procurement of goods and services, clear specifications and service-level agreements, and timely payment.

Effective stewardship of a diverse supply ecosystem requires new skills, including leadership, regulation, and oversight.

Let's be clear—adopting a stewardship role does not involve a reduction in responsibility. There can be no effective public health supply chains without the public sector playing its stewardship role across the policy and operational spectrum. Certain functions remain essential public responsibilities: regulating pharmaceuticals, setting essential medicines policy, defining benefits and services, crafting an overall supply system vision and strategy, overseeing public expenditure, and providing oversight of the health system in general.

Certain supply chain functions remain essential public responsibilities, such as regulating pharmaceuticals and setting essential medicines policy.

## POSITIONING SUPPLY CHAINS FOR THE FUTURE OF PUBLIC HEALTH

Effective supply systems must be able to respond to changes in health priorities, demographics, manufacturing, technology, and financing. In short, they must be *nimble*. Governments typically are *not* nimble. But with appropriate skills, a good understanding of their stewardship responsibility, and a strategic vision of their health sector goals, governments can reengineer their public health supply systems to better serve their mandate and their business. Every country context is different, and no single combination of public and private engagement, nor any single approach, can be applied everywhere. But these principles are universal:

- **Know your business.** It is public health. The bottom line is saving and improving lives, which should be as powerful a motivator for rethinking supply systems as profit is in the commercial sector.
- **Focus on what only you can do.** Concentrate on your core competencies to be a steward (versus a provider) of public health.
- **Learn from the commercial sector.** Ensure leadership at the highest levels for rethinking your supply system, creating change, and pursuing continuous improvement.
- **Embrace diversity.** Identify and leverage the ecosystem of supply chain actors from the public, private, and semi-private sectors to get the job done well and efficiently.

Effective supply systems must be nimble—they must be able to respond to changing development contexts.

Business leaders the world over recognize that cost-effective supply chains are essential and have given them the strategic vision and operational resources they require. Their success can be seen in the manufacturing, agriculture, technology, pharmaceutical, and retail sectors and can be measured on the bottom line. The same techniques that have achieved so much in commerce can be applied by public-sector stewards to the business of public health, strengthening supply chains that will lead to better health and better lives for men, women, and children.

## Notes

**Peer Reviewed**

**First published online:** 2014 Oct 29
